# DEPRESSIVE SYMPTOMS MEDIATE THE LONGITUDINAL ASSOCIATION OF RELIGIOUSNESS WITH SLEEP HEALTH AMONG OLDER ADULTS

**DOI:** 10.1093/geroni/igae098.2549

**Published:** 2024-12-31

**Authors:** Divine Domingo, Tuo Yu Chen, Philile Mgabhi, Kian Lee Lim

**Affiliations:** Taipei Medical University, Taipei, Taipei, Taiwan (Republic of China); Taipei Medical University, Taipei, Taipei, Taiwan (Republic of China); Taipei Medical University, Taipei, Taipei, Taiwan (Republic of China); Taipei Medical University, Taipei, Taipei, Taiwan (Republic of China)

## Abstract

Although previous research shows that religiousness is significantly associated with better sleep health, their underlying mechanisms are unclear. We examined whether psychological distress and healthy behaviors mediated the association of baseline religiousness with multidimensional sleep health at 4-year follow-up among community-dwelling older adults. Data were from the Taiwan Longitudinal Study on Aging (2011-2015; N=2182). Multidimensional sleep health was conceptualized using the SATED model (satisfaction, alertness, timing, efficiency, duration; total score: 0-5), with a higher score indicating better sleep health. Religiousness was operationalized using the average frequency of five religious activities (praying, studying the sutra or bible, going to temple or church, watching or listening to religious programs, and making donations for religious purposes). Mediators at baseline included depressive symptoms (the Center for Epidemiological Studies-Depression Scale), self-reported frequencies of exercise and drinking, and number of cigarettes smoked per day. We used PROCESS macro to test the mediational relationship adjusting for age, sex, education, and chronic conditions at baseline. The results from bootstrapping showed that baseline depressive symptoms significantly mediated the effects of baseline religiousness on multidimensional sleep health at follow-up (indirect effect: B=.01, SE =.004, 95%CI=.0004-.02). Exercise frequency, drinking frequency, and number of cigarettes smoked per day at baseline were not significant mediators. Our finding provided further insights on the link between religiousness and multidimensional sleep health by showing that religiousness was associated with better sleep health through decreased depressive symptoms but not by increased exercise frequency, reduced drinking frequency, and fewer number of cigarettes smoked per day.

